# Modeling of Outpatient Prescribing Process in Iran: A Gateway Toward Electronic Prescribing System

**Published:** 2014

**Authors:** Maryam Ahmadi, Mahnaz Samadbeik, Farahnaz Sadoughi

**Affiliations:** a *Department of Health Information Management, School of Health Management and Information Sciences, Iran University of Medical Sciences, Tehran, Iran.*; b *Department of Health Information Technology, Lorestan University of Medical Sciences, Khoramabad, Iran. *

**Keywords:** Electronic prescribing, Medication prescription, Process analysis, Model, Workflow

## Abstract

Implementation of electronic prescribing system can overcome many problems of the paper prescribing system, and provide numerous opportunities of more effective and advantageous prescribing. Successful implementation of such a system requires complete and deep understanding of work content, human force, and workflow of paper prescribing. The current study was designed in order to model the *current business process of *outpatient prescribing in Iran and clarify different actions during this process. In order to describe the prescribing process and the system features in Iran, the methodology of *business process modeling and analysis *was used in the present study. The results of *the process documentation *were analyzed using a conceptual model of workflow elements and the *technique of modeling “As-Is” business processes*. *Analysis of the current *(as-is) prescribing process demonstrated that Iran stood at the first levels of sophistication in graduated levels of electronic prescribing, namely electronic prescription reference, and that there were problematic areas including bottlenecks, redundant and duplicated work, concentration of decision nodes, and communicative weaknesses among stakeholders of the process. Using information technology in some activities of medication prescription in Iran has not eliminated the dependence of *the stakeholders* on paper-based documents and prescriptions. Therefore, *it is necessary to implement* proper system programming in order to support change management and solve the *problems in the existing *prescribing process. To this end, a suitable *basis should be provided for *reorganization and improvement of the *prescribing process *for the future electronic systems.

## Introduction

Health care systems are under tremendous pressures owing to the demographic changes of population and the subsequent demand for health care. Accordingly, due to the *society's trend toward *medication therapy, the number of medication prescriptions and drug items has steadily increased in most countries and the society has become much more dependent on the prescribing, dispensing and processing system of medication prescriptions (1-6).

Iran is one of the most populous countries in the Middle East (7) and about 85 million *prescriptions are issued annually for all insured patient* in this country (8). Previous studies on paper prescribing in Iran have revealed problems such as preventable medication errors (9,10), transcription errors (11,12), polypharmacy (13.^14^), inappropriate prescription of antibiotics and injectable form of drugs (15-22). Moreover, these studies *have emphasized* *on* the impact of computerized prescription system on reducing medication error and improving patient safety (9, 10).

*Nowadays, limitations and problems *of the *paper*-based *prescription* system and remarkable advantages of information and communication technology have led to initiate new technologies and electronic systems in prescribing as the final solution to eliminate deficiencies of paper prescribing (23-32).

However, it should be noted that there is a strong *relationship *between information technology and business process (33, 34), and inappropriate application of information technology in wrong processes might lead to organizational defeat and unintended consequences of information technology such as unfavorable workflow or untoward changes in communication patterns and practices. Also, improper automation intensifies the *speed of previous errors incidence *and generates new kinds of errors (35-37). Successful implementation of an electronic prescribing system requires complete and deep understanding of the work content, *manpower, *and workflow of paper prescribing. Therefore, it is essential that workflow patterns related to baseline conditions of current prescribing be analyzed. Achieving a thorough *understanding of *the current paper system will prevent any unwanted outcomes due to application of modern technologies that may not be matched with workplace (38-43). 

Since health systems have complex business processes involving several departments with multiple workflows, role definitions, objectives and fiscal interest (44, 45), some researchers have studied critical assessment*, utilization of workflow analysis*, and clinical processes modeling (28, 33, 38, 40, 41, 46-54). A *number of these researchers *have utilized different techniques for process *modeling and workflow analysis of *electronic prescribing (24, 33, 34, 38, 41, 46, 47, 49). *Among these techniques, *Unified Modeling Language (UML) has been more popular as an effective language for *Business Process Modeling *(BPM) of *electronic prescribing* systems (33, 49, 55) and manual prescription writing (38).

The transition to e-prescribing is not a single-step process (42, 56) and, in general, the main actions and steps in the process of prescribing remain the same with advancing computerization level (47). Since the failure to recognize the realities of the current manual process could drastically impact the adoption and successful implementation of future electronic systems, *it is necessary that* the current system be analyzed in the first stage of automation of the prescribing process (38). The current business process modeling and analysis provides a visual snapshot to *acquiring a *general knowledge of the workflow, business processes, activities and actors, and also paves the ground for extracting new system software requirements, implementing actions automatization, and changing management (33, 35). 

Hence, the objective of this study was to analyse and model the current outpatient prescribing process in Iran as well as to clarify the different actions taken during this process. The results of the study can provide a suitable opportunity to reengineer and improve the process of designing future electronic prescribing systems. 

## Experimental

In this study, the Business Process Modeling and Analysis (BPMA) methodology (57) was used to demonstrate the process and system characteristics of the prescribing system in Iran since this modeling technique is a well-established approach used to gain understanding and systematic appraisal, and to identify areas of improvement of a business process. Also, this methodology is now widely utilized for non-software process modeling (52). The business process analysis consists of the following three phases that were carried out in sequence:

Phase I: Scope setting (methods and materials).

Phase II: Data collection, process documentation and analyses framework (methods and materials).

Phase III: Process analysis, bottlenecks identification, discussion and recommendations development (57) (results, conclusion and discussion).

The study method is explained in the first and second phases, and the results, discussion, and suggestions are stated in the third phase.

*Phase I: Scope setting*

The process model of medication management and also the definition of electronic prescribing were used for defining the project scope of this study. The process model of medication management is proposed by Bell *et al.* for evaluating prescribing systems that encompass either handwritten or electronic prescribing. Central to the model are the five major activities involved in medication management: prescribing, transmitting, dispensing, administering, and monitoring (58). Also, the term e-prescribing refers* to *the transmission of prescription or prescription-related information between a prescriber, dispenser, insurance organization or health plan, performed either directly or through an intermediary (59).

Consequently, the project scope was defined from the medication prescribing to the reimbursement claim process, which including three major activities of *Bell's Model* (prescribing, transmitting, dispensing) and *claim reimbursing. *Moreover, only the business process of medication prescriptions related to the outpatient patients covered by Social Security *Insurance* Organization and Medical *Services Insurance* Organization were included in this scope, since based on Article 38 of the *Fifth* *Development Plan Law of Iran, the Iranian Health Insurance Organization *should be formed through the incorporation of all Iranian insurance organizations into the Medical *Services Insurance *Organization *that will *provide public coverage to all members of the society. In addition, the Social Security Insurance Organization was also included in the scope of this study on account of being the largest social insurance institution in the country. 

*Phase II: Data collection, process documentation* *and analyses framework*

A qualitative data collection approach was chosen to provide an in-depth and detailed description of the current *business process of *prescribing in Iran. In this study, the combination of data collection methods including semi-structure interviews, visiting websites of related organizations, examination the *relevant guidelines and publications, *and direct *observation* of *prescription workflow were used. *Methods triangulation was employed by the first author to ensure validation of the data provided during interviews and the conclusions from each of the methods were the same, then validity is established. Also, the research team was independent of the prescribing process.

To initiate construction of workflow model and UML diagrams for outpatient prescribing, the ﬁrst author interviewed semi-structurally *with informants from* the *Office* for *Narcotics and Drug Supervision and Evaluation* of Drug Deputy affiliated to the Food and Drug Organization, *staff investigating and verifying outpatient prescriptions in *Social Security Insurance Organization and Medical Services Insurance Organizations, and also members of *board of directors* of the *Iranian General Practitioners' Association* (*IGPA*) and Iran Pharmacists Association (*See *Table 1). The study involved three types of *sampling methods; *convenience, *purposeful and snowball *sampling. The ﬁnal sample size of n=23 was deﬁned by the point when data saturation occurred and no new explanations emerged. All participants provided written informed consent to participate in the study.

**Table 1:** Interviewees by affiliation

***Number of*** ***Informants***	**Affiliation**
3	Food and Drug Organization (Office for Narcotics and Drug Supervision and Evaluation)
3	Social Security Insurance Organization (staff investigating and verifying outpatient prescriptions)
5	Medical Services Insurance Organizations (staff investigating and verifying outpatient prescriptions)
5	*Iranian General Practitioners' Association* *(IGPA)* *(*members of board of directors)
7	Iran Pharmacists Association *(*members of board of directors)
23	Totals

The interviews were conducted with the aid of a semi-structured interview guide. *The interview guide was prepared based* on series of questions about prescribing workflow walk-through (60) and expanded according to predefined project scope. The interview guide was pretested with a Vice-Chancellor for Food and Drug Affairs to verify face-validity and no modifications were deemed necessary. Data from the pilot interview was not included in the final analyses. In order to increase issues relating to authenticity, accuracy and keep confidentiality of the interviews, we tried to define a tranquil place far from worry of the workplace for interview so that entrance and exit of the clients and telephone ring don’t bother the interviewees and disorder the session. The interview took between 45 and 55 minutes and all session’s times were recorded by two electronic systems in order to prevent potential problems.

All the interviews were digitally audio taped and transcribed verbatim. In order to ensure authenticity of the rewritten conversation, the said texts were revised and confirmed by the interviewees. The recorded interviews were then analyzed using content analysis and coded according to the predetermined categories, which related to the *main elements of conceptual model* of workflow (actors, actions, outcomes, artifacts, aggregation, context, temporality) (61), *components* *of* business use Case diagram (business actors, business use case, association relationship, *include* and *extend relationships) (*62*,*^63^*),* *components* *of* activity diagram (actions, decision point, transaction, *join* nodes or *fork* nodes, swimlane, *initial and end state) (*64*) *and defective areas of prescribing process steps.

The results obtained from content analysis of collected data were depicted by the conceptual model of workflow elements (61) and the *technique of modeling “As-Is” Processes* (57). *The business process modeling was carried* out through use case and activity diagrams of UML. Business use Case diagram was drawn using IBM Rational Rose Version 7.0.0 software since it supports UML v2.1 modelization, and has the capacity of demonstrating business use case and business actor. As it is *not* possible to c*hange* the *font size* on *swim lanes *in the Rational Rose, the activity diagram was drawn by the open source software Argo-UML v0.34 supporting all *standard UML 1.4* diagrams. 

## Results

*Phase III. Process analysis*

**Figure 1:**
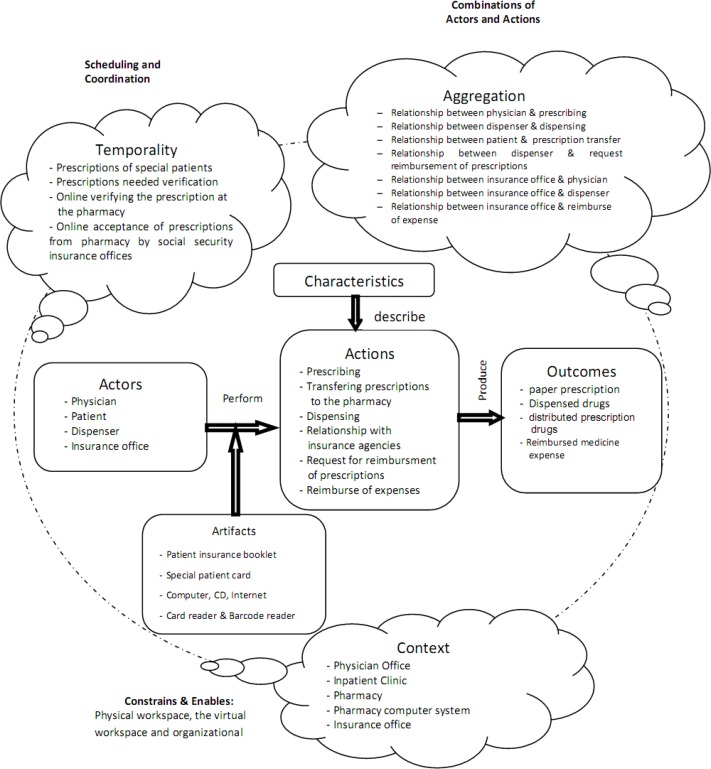
The conceptual model of workflow components for current prescribing process in Iran.

1. The Analysis of the Prescribing Process Using Conceptual Model of Workflow Elements. Successful implementations of e-prescribing systems and the precise depiction of UML diagrams require a deep understanding of the main elements of prescription-writing workﬂow (38). However, there are different approaches toward definition and evaluation of workflow (23, 65, 66); therefore, we used the conceptual model of workflow elements to define the basic workflow elements of the current prescribing process in Iran (Figure 1). The model of elements defining workflow developed by Unertell *et al.* is grounded in the systematic literature review, and is composed of two levels: pervasive and specific.
2. The pervasive level includes three components that apply throughout specific elements of the workflow: context, temporal factors and aggregate factors.
3. The specific level is composed of actors, artifacts, actions, characteristics, and outcomes (61).

*Analysis of the prescribing process using the* technique of modeling “as-is” processes

*Modeling as-is business processes* provides a foundation for defining business activities and improves business processes. In recent years, UML has been highly regarded as an effective technique of modeling business processes and has been recognized as the best way to analyze processes and describe system characteristics using graphical techniques (40, 52, 67), while being comprehensible for people lacking scientific and technical skills (68). Therefore, UML activity and use case diagrams were used to analyze the prescribing process in this study.

*Business process modeling of medication prescription in iran using business use* *case diagram*

**Figure 2:**
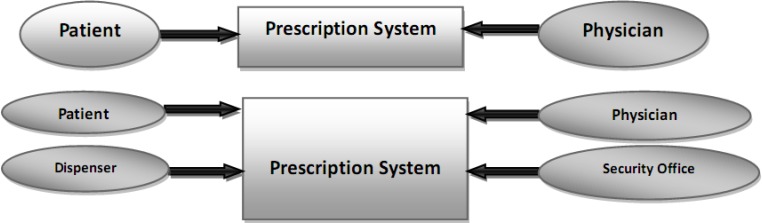
The context diagram of prescribing system in Iran

In first, the context diagram was used in two levels of abstraction in order to find actors, and then the related use cases were recognized according to what people want from the system (Figure 2). 

Use case diagrams *are *the *starting point for* other UML diagrams, and *in spite of their simplicity*, they provide a general overview of the analyzed process. The *use case* model contains a set of use cases, actors, and their relationships extracted from collected data. An actor defines a coherent set of roles that users of an entity can play when interacting with the entity, including human users and other systems. A use case represents the steps in a specific business function or process. The «include» or «uses» relationship indicates that the path of one use case is included in another. The «extends» allows to modify the behaviour of the base use case and shows that a use case provides additional functionality that may be required in another use case (62, 63). 

**Figure 3:**
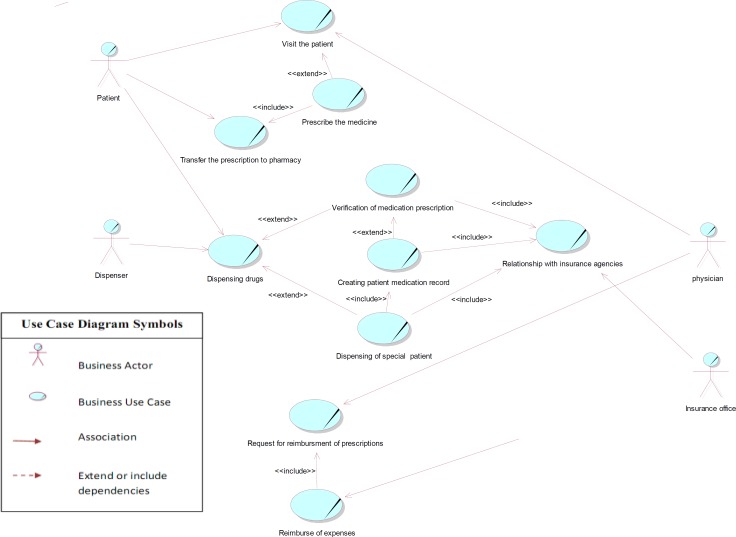
Use case diagram of the prescription process in Iran

As the use case diagram for the current prescription process indicates in Figure 3, there are six main use cases (including prescribing the medicine, transferring the prescription to pharmacy, dispensing drugs, relationship with the insurance organization, request for reimbursement of prescriptions), and four main actors (including physician, patient, dispenser, and insurance office) in this area. The dispenser actor means *pharmacy*, *dispensing pharmacist* or pharmacy technician, and the insurance office consists of the *prescribing investigators or verifier staff in *insurance organizations.

In this diagram, the relationship between use cases of patient visit and prescribing was shown by «extend» connection, because visit use case is complete even without prescribing use case, and the visit might be done without any medication prescription. The use case of prescription is, therefore, optional. In this analysis, we only considered visits that entailed prescriptions. 

Considering that in the process of dispensing, the circumstances might be different, we also used «extend» relation for illustrating the relationship between the dispensing under different circumstances (for instance dispensing of special patients' prescriptions and verification of medication prescriptions), indicating that the dispensing process in these conditions differs from that in general prescriptions.

Nowadays, dispensing medication to special patients (including those being treated for complex disorders such as haemophilia, multiple sclerosis and thalassemia, as well as transplant and kidney dialysis patients) requires creation of a medication record in the database of special patients, only to be performed by personal *encounter *at related insurance offices. Hence, the «*include*» relationship was used for depicting the compulsory connection between use cases related to the dispensing of medication to special patients, creating patient medication record, and also the relationship with insurance agencies. Also, *personal attendance at *insurance offices and creation of a medication record is initially required for *internet verification of some other prescriptions *as their medication are *above the defined cost limit *pricing. In following encounters, however, it is possible to verify the medication from the pharmacy through the internet. On the other hand, pharmacies are eligible to verify *prescriptions *under the *defined cost limit* pricing online without having to create a medication record. Therefore, «*extend*» relationship was used to represent the conditional relationship between use cases of verification of medication prescription and creating patient medication.

*The base use case of *reimbursing the expenses includes the functionality of request for reimbursement use case, as prescription investigation in order to reimburse the costs can only be done after receiving all necessary documents for related insurance agencies (including a list of prescriptions, *electronic files for prescription records*, physical prescriptions, and paper reimbursement claims). This is the reason why “include” obligatory relation was used between these use cases. The diagonal line on use cases and actors refers to business use case modeling with UML2. 

*Business process modeling of prescription in iran using activity diagram*

**“**Activity diagram is typically used for modeling the logic captured in a specific use case in a use case diagram” (64). An activity diagram, also referred to as a workflow diagram, employs traditional flowchart techniques to model workflow, information exchange, and business processes (33, 38, 69-71). This type of diagram is suitable for depicting the dynamic nature of the prescription process. Activity Diagram is used to model a specific actor's workflow within the entire system and shows all potential sequence flows in an activity. The Activity Diagram is comprised of the model elements including, actions, decision point, transaction, *join* nodes or *fork* nodes, swim lane, *initial and end* *state (*64*).*

**Figure 4:**
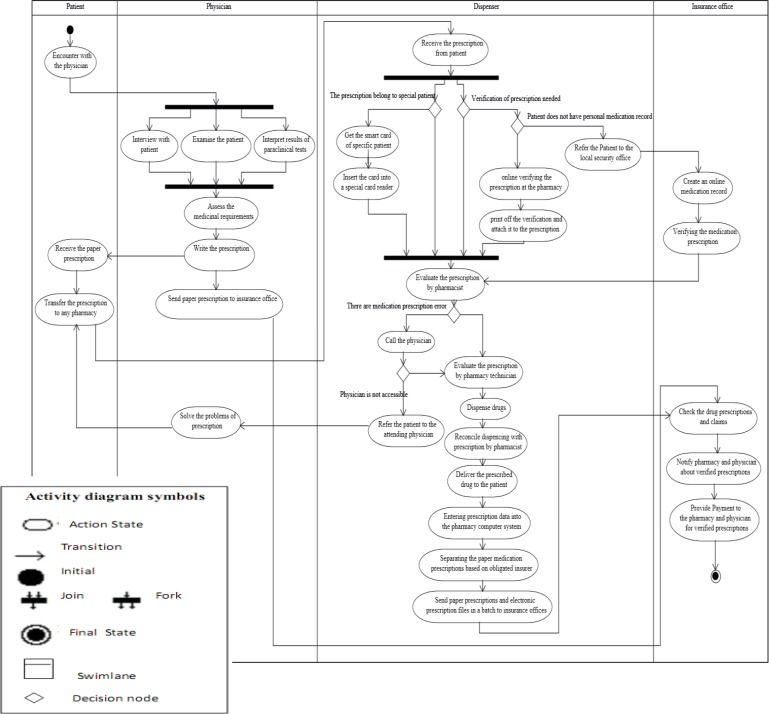
Activity diagram of prescription process in Iran

The activity diagram of the prescription process in Iran (Figure 4) indicates the order of activities, tasks and how they are assigned to different actors (roles) in this business process. Prescription process starts with the activity initial node of the patient-physician encounter and ends in the activity final node of the *reimbursement *for dispensed *prescription medications*. The activity diagram displays four swim lanes, representing the roles of the patient, the physician, the dispenser and the insurance office (sequencing). These swim lanes contain the sequence of *activities* performed by the *actors.*

*The fork node at the beginning and the joint node at the end have *been used in order to show *simultaneous and parallel activities *associated with the use case of visiting the patient in the physician swim lane column (including patient interview, patient physical examination, and *clinical results interpretation)*, and also to depict parallel activities associated with the use case of dispensing in the dispenser swim lane column (including dispensing for special patients and medication prescriptions requiring insurance verification). The reason is that a fork node has one input flow and several output flows, while a join node has several input flows and one output flow. Using decision nodes, the change in the work content of dispenser activities has been demonstrated based on conditions resulting from different *types* of medication prescription (including those belonging to special patients that require insurance verification and the creation of medication records in insurance offices) and also evaluation of prescriptions by the pharmacist (including problematic prescriptions, and prescriptions that require the *pharmacist's accessibility to* the patient). 

*Phase III: Bottlenecks identification*

Qualitative Content analysis based on defective areas category and also process analysis to address problematic areas (*e.g.* redundant and duplicated work, concentration of decision nodes, and communicative weaknesses among stakeholders) led to detecting bottlenecks of the prescription process in Iran. These bottlenecks then categorised by research team based on identified use cases and swim lanes of the current prescribing process models in Iran. The results of the bottlenecks Identification phase is presented as below**:**

- In the use case of prescription and the swim lane of the physician: The physician prescribes medications without* considering the patient’s *financial status, *medication coverage by *the *patient's insurance plan*, Iranian *pharmacopoeia of insurance* coverage, and patient preferences. This kind of prescription increases the *patient’s dissatisfaction and leads to generation of prescriptions that are not covered* by any *insurance *plan.

*Paper*-*based prescribing* prevents evaluation of medication-specific factors and patient-specific clinical and non-clinical factors, access to external resources, and the use of clinical decision support systems for the medication *prescription* process. Therefore, *warnings* about drug interactions, contraindications, medication allergies and *repetitive medication do not appear at the time of prescribing. Also, *it is not *possible to calculate dosage automatically according* to *age*, gender, weight, health history, and so forth.

Prescription does not have a unique prescription number for *identification and tracking. *The *volume and page number of *the patient’s insurance booklet is the only prescription identifier that has been used in *pharmacy software* systems over the years. However, Social Security Insurance Organization has recently started recording the 18-digit serial number and barcode (composed of the patient’s National Identifier Number, insurance branch code, *volume number and page number*) on each page of the Social Security Insurance booklet in order to provide the required *preparation for electronic health record implementation.*

There is no data element about the *number and duration of refills *on medication prescription. 

The physician cannot review and track the paper prescription after the drug is picked up by the patient. Therefore, it is not possible to modify and cancel the existing prescription, or discontinue a specific medication, if the *patient's condition changes.*

- In the use case of dispensing and the swim lane of the dispenser:

All paper prescription data must be entered in the pharmacy software system to provide the *electronic file containing prescriptions* and dispensing information to be sent to insurance offices. This causes a bottleneck that slows down the process of dispensing prescriptions and imposes extra work on the pharmacy.

*The patient's insurance number *is the most important identifier of the patient in the pharmacy software system, while it is *not a unique patient identifier*. In this regard, the Social Security Insurance Organization has withdrawn the old insurance booklets that did not contain the *National Identifier Number*, and has replaced insurance booklet serial numbers with this unique number. 

Pharmacies do not have a *unique* national *identifier *so the Iranian *Food and Drug Organization of the *Ministry of Health *and *insurance organizations use different identifiers to recognize pharmacies.

A fundamental difficulty of this process is the multiple drug codes used by Food and Drug Organization of the Ministry of Health and Medical Education of Iran [Iran Registration Code (IRC) and European Article Number 13 (EAN-13)], insurer organizations formal five-digit medication code, and Saman Salamat Company (*16 digit authentication code of drugs)*. IRC is a common number for pharmaceutical products stored in the data bank of the Division of Pharmaceutical and Narcotic Affairs of the Drug Supervision and Evaluation Office, from which all information about pharmaceutical products can be obtained.

Creation of a medication record is initially required for *internet verification of prescriptions *with medications that are *above the defined cost limit *pricing.

*Although provided opportunity for online verification of prescriptions* from the pharmacy, the poor quality of the internet connection in some cases has caused wandering of the patients to have their medication prescriptions verified. 

Special *patient smart cards *are only used for *dispensing each specific patient's medication* based on the defined medication ratio, while this card *has been designed for use through the* prescription processes.

Refilling and renewal of covered medication prescriptions is not possible through pharmacy and without referring to the physician. If the number of refills is indicated on the prescription, the total *price for the renewed prescription will have to be paid *by the patient *without taking insurance coverage into consideration.*

Phone call is the only communication channel between pharmacists and physicians; therefore, the patient will be referred to the physician if *accessibility to physician is not* possible through this channel when required. This shows the *weakness of communication *between pharmacists and physicians. 

The physician cannot be *notified* of the filled or refilled prescriptions in the expected time course, and therefore it is not possible to *identify the unfilled prescriptions*. 

In the swim lane of the dispenser, decision node concentration is seen in activities of medication prescriptions verification, dispensing of prescription for special patients, evaluation of prescription by pharmacists, and *pharmacists’ communications with physicians*. The use case management of dispensing is therefore difficult because of the *unpredictability of the activities resulting from these nodes.*

- In the use case of prescription reimbursement and the swim lane of the insurance office:

*Despite automation of *some activities in the prescription process such as entering the prescription data in the pharmacy software system and the *online claiming system of *Social Security Insurance Organization, the prescriptions investigation in insurance offices is still done after receiving physical prescriptions.

Insurance offices have different policies regarding receiving, investigating and verifying medication prescriptions, and identical services are provided differently by different health insurance organizations.

Paper prescriptions are legally the only valid documents for dispensing and investigating medication prescriptions, and using electronic prescriptions is legally forbidden. This legal limitation has led to rework loops, redundant and duplicated work in this process. 

## Discussion

Results of the process analysis indicated that main use cases and actors of the prescribing process in Iran were similarly the *same with *other studies in this field (28, 33, 38, 47), *as they are common in* four main actors (including Prescriber, patient, dispenser, and reimbursement agency) and four main use cases (including prescribing the medicine, transferring the prescription to pharmacy, dispensing drugs and reimbursing* claim)*. Moreover, a unique identifier was not used for prescriptions, patients, prescribers, dispensers and pharmacies in the current prescribing system, while each of these entities should have a single unique identifier, and the development of unified identifiers is an essential prerequisite for the rapid and safe development of integrated prescribing systems (51) as suggested by other researchers (5, 49, 58, 72). In this process, the *activities of prescribing, *transferring the prescription to pharmacy and dispensing were performed manually, while activities of submitting *reimbursement claims*, dispensing medication for special patients, verifying prescriptions, and reimbursing the costs of dispensed medication are carried out *semi-automatically*. However, using information technology in some activities of medication prescription has not eliminated the dependence of insurance organizations on paper-based documents and prescriptions (73). Meanwhile, inconsistencies* in the policies and practices used by different insurance organizations regarding medication prescription has been pointed out in this research and other studies done in Iran (*74*-*77*).*

E Health Initiative (EHI) has outlined six different graduated levels of e-prescribing from basic reference systems to advanced systems demonstrated in a pyramid. Each level covers more functionalities than previous one. “The levels of the pyramid are: “1. Electronic prescription reference only, no prescribing capability; 2. Stand-alone prescription writer, with no medication history or supporting data; 3. Addition of basic supporting data, such as allergies, demographics, and formulary information, which can be used by the system to generate alerts; 4. Medication management, long-term tracking and monitoring of each patient’s active medications; 5. Connectivity among practices, pharmacies, payers, pharmacy benefit managers(PBM’s), intermediaries, and patients; 6. Integration with a more complete electronic health record” (78). Therefore, Iran is placed in the first graduated level of sophistication based on this model. Moreover, since drug *information*, *dosage calculation and pharmacopoeia *are accessible as free text or digitally but are not *automatically* displayed *when prescribing*, *medical error* reduction and *prevention* is *extremely* difficult in this system, although most *commercial e*-*prescribing products* at least provide significant features at levels 2, 3 and 4.

Also, the country is in the first step of the five-stage model adopted by e-Health Observatory in order to subdivide e-prescribing functionality. This five-stage model includes zero stage of paper prescription environment to fifth stage of complete implementation of electronic prescription and link with the electronic medical record (EMR). In this model, some new processes do enter the workflow with increasingly sophisticated e-prescribing systems (42). Hence, *Iran’s prescription system has crossed from *stage zero of *completely *paper-based prescribing environment to stage one due to the fact that many applications such as information systems for health care organizations have been used in this domain. Because*, it's* *suggested that* the presence of information technology utilization in the medical centers can facilitate the positive pharmacotherapy interventions, saving lives and minimizing health care costs (79). However, current softwares are not* in compliance with *information exchange standards and *decision support *systems (73). 

*Phase III: Recommendations development*

According to the research results, the following suggestions are proposed for solving the problems of the current prescribing process in Iran and paving the way for implementing electronic prescribing system:

- Using unique identifiers for patients, prescriptions, medications, and pharmacies.
- *Extending *coverage to all members of the society by the Iran Health Insurance Organization and *creating *an *integrated payment system.*
- Correcting rework loops and eliminating redundant and duplicated works in this process.
- Considering electronic prescribing as a part of electronic health project.
- Establishing a centralized national prescription database.
- Connecting required databases such as medication, demographic, and *decision support system databases *to the central prescribing database.
- Defining a proctor to implement and preserve the centralized national prescription database. Since the Statistics and Information Technology Office affiliated with the Ministry of Health and Medical Education has the responsibility to design and establish electronic health records, we suggest that the responsibility of implementing the electronic prescribing system be assigned to this office as well.
- *Approving the ne* *cessary laws mandating electronic prescribing*, *legitimating electronic signature *and accepting electronic versions of prescriptions as legal documents for reimbursement.
- Converting all paper artifacts used in medication prescribing to electronic artifacts.
- *Defining* *all stakeholder information requirements* for the prescribing process.
- *Using the capacity of *the national smart card for electronic health records, and considering health card as a component of the national smart card.
- Merging and integrating the treatment smart card and the smart card of Medical *Services Insurance* Organization with the national smart card.
- Providing the opportunity to move toward a paperless environment for activities of *medication *prescribing, transmitting, dispensing, claiming, reimbursing and refilling via electronic connectivity between physicians, pharmacists and insurance organizations.
- *Providing easy access to the *information of the formulary, medication history and patient eligibility at the time of prescribing.
- *Preparing *free hardware, software and technical support, or considering the appropriate mixture of incentives to attract related stakeholders to e-prescribing.
